# Effects of a Sprinkler and Cool Cell Combined System on Cooling Water Usage, Litter Moisture, and Indoor Environment of Broiler Houses

**DOI:** 10.3390/ani13182939

**Published:** 2023-09-16

**Authors:** Jonathan Moon, Jan DuBien, Reshma Ramachandran, Yi Liang, Sami Dridi, Tom Tabler

**Affiliations:** 1 Department of Poultry Science, Mississippi State University, Mississippi State, MS 39762, USA; 2 Department of Mathematics and Statistics, Mississippi State University, Mississippi State, MS 39762, USA; 3 Center of Excellence for Poultry Science, Department of Biological and Agricultural Engineering, University of Arkansas, Fayetteville, AR 72701, USA; 4 Department of Poultry Science, University of Arkansas, Fayetteville, AR 72701, USA; 5 Department of Animal Science, University of Tennessee, Knoxville, TN 37996, USA

**Keywords:** broiler cooling, climate change, sprinkler, sustainability, water efficiency

## Abstract

**Simple Summary:**

Water scarcity is an increasing problem facing the global agricultural industry, particularly livestock production. The poultry industry is actively seeking opportunities to reduce the use of water for cooling broiler houses during hot summer months. We investigated a sprinkler system combined with a cool cell system and a cool-cell only system for cooling heavy broiler chickens for two summer flocks. We found the sprinkler/cool cell combination exhibited a higher house temperature, lower relative humidity, and a 64% reduction in average cooling water use. Litter moisture also tended to be lower in the combination system. Findings are similar to previous reported research and offer additional confirmation that sprinklers in conjunction with cool cells maintain litter conditions while reducing cooling water use, thus lessening the threat to the economic and environmental sustainability of the poultry industry and improving its water efficiency efforts.

**Abstract:**

Climate change is a serious challenge to food production around the world. Sustainability and water efficiency are critical to a poultry industry faced with global production concerns including increased demands for high-quality, affordable animal protein and greater environmental pressures resulting from rising global temperatures, flock heat stress, and limits on water availability. To address these concerns, a commercial sprinkler system used in combination with a cool cell system was evaluated against a cool cell system alone for two summer flocks of heavy broilers at Mississippi State University to determine effects of sprinkler technology on cooling water usage, litter moisture, and in-house environments. Environmental data were calculated and recorded throughout the flocks. The combination house exhibited a 2.2 °C (4 °F) increase in daily maximum temperature, lower coincident relative humidity, and a 64% (62,039 L/flock) reduction in average cooling water usage over the cool cell-only house. Litter moisture for the combination house tended to be numerically lower but showed no significant difference at several time points between and across flocks. A combined sprinkler/cool cell system reduced cooling water use by 64% over two flocks compared to a cool cell alone system and decreased in-house relative humidity levels.

## 1. Introduction

Poultry meat represented almost 40 percent of global meat production in 2020 [1]. Poultry is one of the main sources of animal protein due to its universal acceptability, high nutritional value, and health benefits [2]. The growth in global population has pressured the poultry industry to increase its production capacity [3,4,5]. Global meat production doubled from 1980 to 2004 and is projected to double again from 2000 to 2050 [6]. This rapid growth in global meat production puts pressure on water resources because livestock production is a very water-intensive agricultural activity, i.e., about one-third of the total water that is utilized in global agricultural production is assigned to animal production [2]. Broiler chickens are one of the most efficient animals in terms of growth rate and feed conversion ratio [7]. Broiler chicken meat is considered to have a relatively low carbon footprint among all farmed meat products [8]. According to previous research, feed is the largest contributor to climate change impact associated with broiler production [9,10,11,12,13,14,15,16]. However, water scarcity, always a concern in developing countries, is rapidly emerging as a global concern [17,18]. Estimates are that by 2025, half the world’s population will be living in countries facing considerable water stress or scarcity issues [19,20,21]. A changing environment threatens water security and can affect the future of poultry production, making water conservation efforts critical to the sustainability of the poultry industry. Climate change is creating new challenges such as increasing the earth’s temperature by 0.2 °C per decade with significant fluctuation in the amount and distribution of rainfall, resulting in longer and more intense heat waves and increasing global water scarcity concerns [2].

Commercial broiler chickens are raised in specially designed houses capable of maintaining an environment that allows for optimum performance even during long periods of high environmental challenges. These houses are the result of decades worth of research to determine the correct combination of cooling and ventilation [22,23,24]. In recent years, consumers and the poultry industry have placed increasing emphasis on raising chickens in a more sustainable manner. Water conservation is a major emphasis for the poultry industry today, as sustainability and global food security issues challenge the industry to meet consumers’ demands for safe high quality affordable meat protein and lessen the industry’s overall environmental footprint [16].

Climate change and heat stress are additional challenges to sustainable poultry production. Water is crucial in poultry production not only in bird consumption but also in alleviating heat stress during cooling periods [25,26]. Evaporative cooling pad systems, while effective at reducing the temperature of the air entering the poultry house, often result in excessive relative humidity levels of 80% or higher in the house, require large volumes of water [27], and negatively affect the ability for broilers to dissipate heat through evaporative respiration during periods of high environmental temperatures [28,29,30,31,32]. Under normal conditions, broilers typically achieve heat dissipation primarily through respiratory evaporation [33,34], which is severely hindered by high in-house humidity levels. High in-house humidity level is a known factor that negatively affects litter quality and thereby, animal welfare [35,36,37,38,39]. High environmental temperatures combined with high in-house humidity levels can create life-threatening conditions to broiler chickens as they near targeted market weight and age.

Animals dissipate body heat through two mechanisms, i.e., sensible heat dissipation and evaporation (latent heat dissipation). The temperature gradient between the animal and its surroundings governs the sensible heat dissipation. Evaporative cooling pad systems employed in modern tunnel-ventilation poultry houses aim to lower temperature of the surroundings, hence increase the sensible portion of the heat dissipation. In comparison, evaporative heat dissipation from birds is governed by the vapor pressure gradient rather than by temperature differences. As the ambient temperature comes close to the body temperature, evaporative heat flux becomes the only pathway for an animal to dissipate heat to maintain a constant body temperature. Chickens do not have sweat glands but lose heat mainly through panting, with some heat also lost by skin surface evaporation [40]. Directly applying controlled amounts of water onto poultry using sprinkler systems partially wets the birds, allowing direct evaporation of water from the birds’ surface. Water evaporation absorbs heat directly from the body of the birds and the surrounding air, similar to water evaporation from evaporative cooling pads at the tunnel-ventilation air inlet. For water to evaporate from the chicken surface efficiently, it is crucial to maintain a large vapor pressure gradient by keeping the humidity of the surrounding air low. In contrast, evaporative cooling pads, along with high-pressure or low-pressure fogging systems would increase the humidity inside the poultry house and be less able to reduce the effective environmental temperature.

Research has reported efficacy of surface wetting and its effect on body temperature of laying hens [41,42] and broilers [30] under controlled thermal conditions, and commercial production conditions [43,44,45,46]. Sprinkler systems offer water conservation advantages without sacrificing flock performance [31,45,46,47]. However, it is a challenge to incorporate sprinkler systems into the existing evaporative cooling systems which have been in use for more than two decades in the southern United States. As mentioned earlier, surface wetting by sprinklers requires low in-house humidity to function properly. It is necessary to develop a management strategy such as a set point and application schedule in a sprinkler and cool cell combined system to achieve not only cooling water savings, but also satisfactory air and litter quality.

The objective of this study was to develop and evaluate a management strategy under a combined sprinkler and cool cell technology on cooling water conservation, in-house environments, and litter moisture conditions when raising heavy broilers on a commercial broiler farm.

## 2. Materials and Methods

### 2.1. Broiler Houses

The study was conducted at two commercial broiler houses on the Mississippi State University poultry research farm for two summer flocks from May through October of 2020. For the May-placed flock, each house received 13,700 straight run Ross × Ross 708 broiler chicks on day of hatch. For the August-placed flock, each house received 14,700 straight run Ross × Ross 708 broiler chicks on day of hatch. Both flocks were heavy broilers with a final target body weight of 4.42 kg. The two houses were each 13 m × 122 m (42 ft × 400 ft) and equipped with three lines of pan type feeders and four lines of nipple-type drinkers. Both houses were drop ceiling houses and neither house contained ceiling baffles.

Each house contained 15 m (50 ft) of 1.5 m × 15 cm × 0.30 m (5 ft × 6 in × 1 ft) cool cell on each side of the house. Ten 122 cm (48-in) diameter tunnel ventilation fans (Acme Engineering and Manufacturing Corp., Muskogee, OK, USA) were at the opposite end from the cool cells in each house. Each house was also equipped with two lines of commercial sprinklers (Weeden Environments, Woodstock, ON, Canada ) mounted to the ceiling and located 3 m (10 ft) from each sidewall above the two outside feed lines. The sprinkler lines consisted of 19 mm (¾ in) PVC pipe running the length of the house with 275 kPa pressure regulators. Sprinkler spinner heads with flexible droppers of nominal flow rate of 1.3 L/min were located every 6 m (20 ft) down each line and were directly across from one another (e.g., not staggered). There were 20 spinner heads on each line; a total of 40 per house located 2.1 m (7 ft) above the litter.

The evaporative cooling system remained intact in each house. The cool cell system was controlled by a Chore-Tronics 3 (Chore-Time, Milford, IN, USA) poultry house controller. An integrator-developed cooling program using their set point temperatures and run times was followed in the cool cell only house. For the two summer flocks, one house was cooled by the evaporative cooling system only. For this house, the set point temperature on the cool cell pads was 28 °C (82 °F). The tunnel set point temperature was always 3.3 °C (6 °F) above the house set point temperature for any given day.

The sprinkler system was operated in combination with the cool cell system and not as a stand-alone cooling system, although previous research has demonstrated successful sprinkler use in a stand-alone setting [46]. The combination-cooling house was cooled by the sprinkler system as the first stage of cooling with assistance from the cool cell system once house temperature reached 32 °C (90 °F). This was accomplished by modifying the operational settings on the cool cell set point in the poultry house controller program. The cool cell set point temperature was raised to run water over the pads for 15–20 s, but only when the house temperature reached 32 °C. The goal of the cool cell in the combination-cooling house was not to cool the house temperature and increase the humidity as in the cool cell only house, but to prevent the house temperature from going any higher than 32 °C. The two houses were switched between the two flocks to remove any house effect (e.g., the cool cell house on the first flock became the sprinkler/cool cell combination house on the second flock and vice versa). The sprinkler system and the evaporative cool cells were allowed to operate from 9:00 am to 9:00 pm.

### 2.2. Sprinkler System

Both houses were equipped with a low-pressure commercial sprinkler system (Weeden Environments, Woodstock, ON, Canada) capable of three levels of cooling intensity that is explained below. The sprinkler controller was mounted in the control room of each house where the main house controller was located. However, there was no communication between the two controllers. The sprinkler system consisted of two zones, with 20 spinner heads in each zone and one temperature sensor (at bird height) located approximately in the center of each zone near the north side feed line. The brood half of the house containing the cool cells was one zone, and the non-brood half containing the tunnel fans was the second zone. Each zone was operated independently by activating an electronic solenoid valve assigned to that zone depending on the temperature in that zone. As a result, the two zones might run on different schedules and be in different intensity levels at a given time.

Sprinklers in the combination-cooling house and cool cells in the cool cell only house were allowed to operate from d 37 until harvest (d 61) for the first summer flock (Table 1). Cool cells in the combination house were allowed to operate from d 53 until harvest (d 61). During the second summer flock, sprinklers and cool cells in the combination house and cool cells in the cool cell only house were all allowed to operate from d 27 until harvest (d 60).

The three levels of cooling programmed into the sprinkler controller served different functions. The levels recommended by the manufacturer were as follows: Level 1 begins at 1.1 °C above the tunnel set point temperature (TT) and operates for 10 sec every 30 min (Table 2). When sprinkled, birds stand up and release trapped heat between and under the birds. Additionally, upon standing, numerous birds were observed to move to the feeder and drinker lines to eat and drink. Level 2 activates at 2.8 °C above the TT and operates for 20 s every 15 min. It combines getting the birds up to release trapped heat with increased wind chill on the birds from additional tunnel fans operating and increased amount of sprinkler droplets on the heads and feathers of the birds. Level 3 activates at 4.4 °C above TT and operates for 20 s every 5–7 min, depending on conditions, and creates bird surface wetting that allows maximum wind chill because of the nearly constant evaporative cooling of water droplets off the birds and a steady wind speed above 2.5 m/s (500 ft/min). For this study, 8 tunnel fans were running during Level 1, 9 fans during Level 2, and 10 fans during Level 3. Even though the tunnel fan set points were staged 0.56 °C apart, fans 9 and 10 were withheld until the sprinkler system reached levels 2 and 3, respectively. Table 2 lists temperature and run time settings suggested by the manufacturer. Table 3 lists sprinkler and cool cell settings used in the study.

We operated the sprinklers under similar idle times of each cooling level but at temperature settings slightly higher than recommended by the manufacturer (Table 3). In the combination house, the cool cell (CC) was allowed to be wetted for a short duration of 15–20 s at a time when CC setpoint temperature of 32 °C (90 °F) was reached in order to prevent substantial decrease of indoor temperature. The goal is to allow the sprinklers to do as much cooling as possible and use the cool cells only in extreme situations. It is critical to not run the cool cell system too soon, too often, or too long when sprinklers are used. Doing so tends to keep the house too cool and too humid for the sprinklers to be most effective. Ideally, cool cells should not operate before a house temperature of 31 °C is reached for the sprinklers to be most efficient. While this higher temperature may seem frightening to some, it comes with a lower humidity level that is beneficial to house environment, allowing birds to utilize evaporative respiration more efficiently.

### 2.3. Measurements

In-house temperature and relative humidity data were monitored and recorded by an Intelia data collection system (Intelia Technologies Inc., Quebec, QC, Canada) in each house with collection interval of 15 min. Cooling water use by sprinkler and evaporative cooling systems were monitored using water meters containing an electrical pulse output (1 pulse = 3.8 L (1 gal)).

Initial bedding material was kiln-dried pine shavings. However, at the time of the study, the two broiler houses had not received a total litter clean out in over 10 years. Every two years, approximately one half of the litter material was removed from each house to re-establish a litter depth of four to six inches and the remaining litter was evenly spread back out and was used to grow additional flocks. No topdressing of litter was performed between flocks. Chicks were placed directly on old litter material after caked litter was removed and the litter re-leveled. Down time between flocks was 22 d for the first flock and 17 d for the second flock. Litter moisture content was measured by sampling litter at the end of weeks 7 and 9 during both flocks. Litter was collected separately from 16 locations in the cool cell and fan ends of the houses. The eight subsamples from each end (16 total subsamples) were collected from the top 1–2 cm of the litter surface using a round point shovel and thoroughly mixed in a 19 L (5-gal) bucket. From this mixed sample, a 946 mL composite subsample was placed in a plastic bag and transported to the Mississippi State University Chemical Laboratory for moisture content analysis [48]. Production data such as feed conversion ratio (FCR), body weight, mortality, and paw quality data were collected by processor at harvest. However, there were no significant differences in production data; therefore, production data are not discussed in the current paper.

### 2.4. Statistical Analysis

Maximum indoor temperatures of each day (collected every 15-min) for the time period 9:00 am to 9:00 pm (when water cooling systems were operational) were collected and averaged as daily averaged maximum temperature from each house. Coincident relative humidity (RH) values when the maximum indoor temperatures occurred were collected and averaged for this duration. The temperature and RH values from two cooling regimes were analyzed using one-way ANOVA using SAS 9.4 with significance indicated by *p* ≤ 0.05. Litter moisture contents of the two flocks from either cool cell half or the tunnel fan half for weeks 7 and 9 were analyzed separately. Although low replication was an issue in this study, as is always the case with whole-house treatments on commercial poultry farms, results are similar to those reported previously [31,47,49].

## 3. Results

### 3.1. Relative Humidity and Temperature

**Figure 1:**
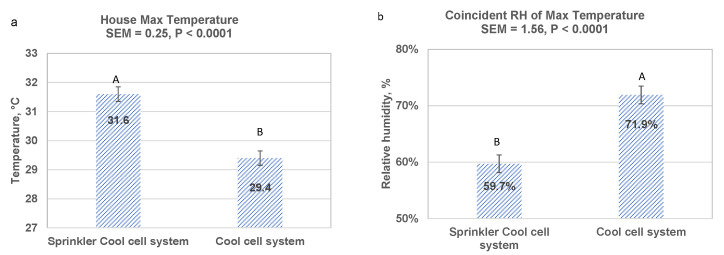
Thermal environment when cooling was in effect to raise heavy broilers during May–Oct 2020. (**a**) Average daily maximum house temperature, and (**b**) average of coincident relative humidity as maximum temperature occurred. SEM: Pooled standard error of mean. Letters A or B showing significant difference.

Maintaining cool cell set point temperature in the combination-cooling house at 32 °C (90 °F) resulted in limited use of cool cells in the combination house. As a result, the daily averaged maximum temperature in the combination house was 2.2 °C higher (*p* < 0.0001) in the combination house than in the cool cell only house (Figure 1a). However, the higher temperature was offset by a 12.2% lower coincident humidity (*p* < 0.0001) in the combination house (Figure 1b). The effect of sprinklers on the in-house environment showed the trend of lower relative humidity and higher temperature in the combination house than those in the cool cell only house during day-time cooling period, consistent with previous studies [31,50].

**Figure 2:**
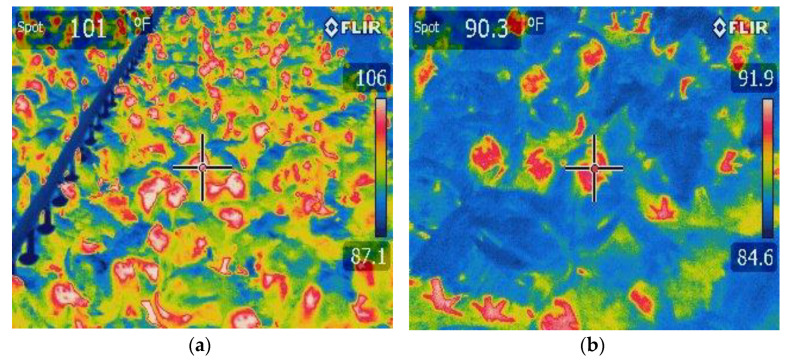
Thermal images showing surface temperature of birds in a combination-cooling house at 58 d before sprinkling (**a**) and after sprinkling (**b**). The wetted surface due to sprinkler operation lowered body surface temperature overall (blue color (**b**)) with most of chickens’ feet and head/neck shown as warm red color.

Even though maximum house temperatures in the combination house were higher (Figure 1a), this should not be equated with actual bird comfort temperature. The direct cooling effect of the sprinklers on the birds, indicated by the lower surface temperature (Figure 2a,b), was effective in facilitating heat release from birds and compensating for higher environmental ambient temperature [30,31,32,33,34,45,51]. High ambient temperatures resulted in use of evaporative cooling pads in the cool cell house and sprinklers in the sprinkler/cool cell combination house.

### 3.2. Water Used for Cooling

**Figure 3:**
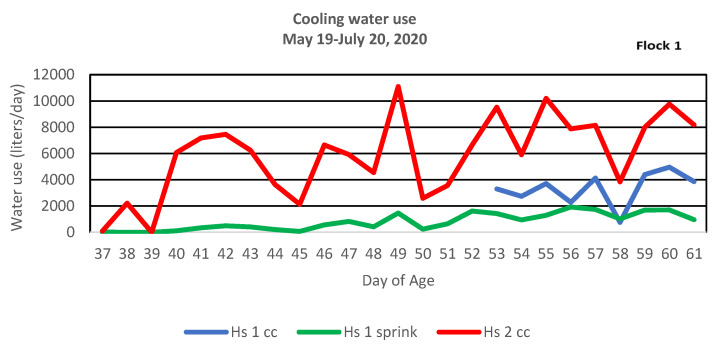

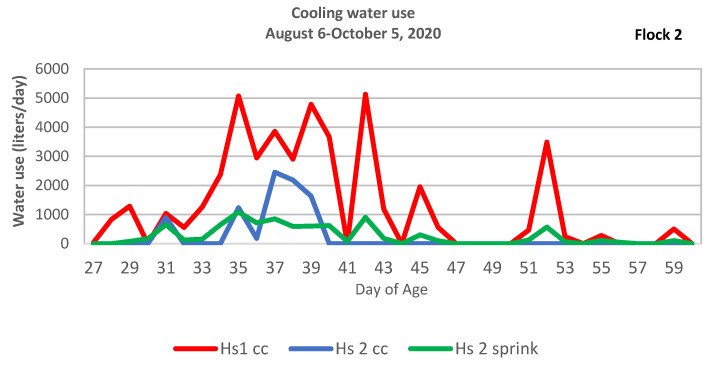
Daily cooling water use of sprinklers and cool cells for two flocks in two houses from May to October 2020. House 1 cool cell (Hs 1 cc), House 1 sprinkler (Hs 1 sprink), House 2 cool cell (Hs 2 cc), and House 2 sprinkler (Hs 2 sprink).

Water is essential for a variety of physiological functions and the productive performance of chickens. However, with recent uncertainties exacerbated by climate change, plentiful water availability is no longer a certainty in many locations and water scarcity is becoming a major global concern. Several factors affect the daily water requirement for poultry: e.g., housing conditions (temperature, lighting program and intensity, etc.), performance level, and feeding-related factors (type and ingredients) [2]. A major benefit associated with the sprinkler system is the potential cooling water savings compared to a cool cell only system. Daily cooling water use for each house is reported per flock in Figure 3. Cumulative cooling water for each flock is reported in Table 4. Water usage in the sprinkler/cool cell combination house demonstrated water savings that averaged 64% over the two summer flocks in comparison to the cool cell only house. These savings are in close agreement with [31] where savings of 67% were reported and [50] where savings of 58% were reported. The greatest water savings were observed on days when only sprinklers were in use in the combination house while evaporative cooling pads were being used in the cool cell house.

### 3.3. Litter Moisture

**Figure 4:**
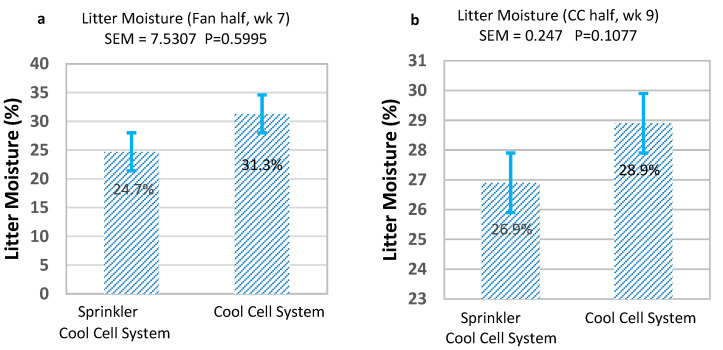
Average litter moisture contents over two flocks. (**a**) fan half of houses in week 7; (**b**) cool cell half of houses in week 9. SEM: Pooled standard error of mean.

We saw no significant effect of sprinklers on litter moisture contents in either the fan half (Figure 4a) or cool cell half (Figure 4b) of the house at either week 7 or week 9 of the flocks. This is in agreement with findings from [31] who reported no significant effect by sprinklers on litter moisture content. However, it does not agree with research by [50] who found moisture content differed with a two-way interaction between growout × sprinklers (*p* = 0.002), with slightly drier litter in the sprinkler house. A weaker relationship was also found by [50] when sprinklers were considered as a main effect (*p* = 0.046), where litter moisture was slightly lower in the sprinkler houses. In the current study, we did see slightly drier litter in the sprinkler/cool cell combination house. Litter moisture content is a result of excreted moisture, normal drinker spillage, leaking drinkers, and water evaporation into the air [39,51]. The potential lower relative humidity and higher temperature in the sprinkler/cool cell combination house, hence higher water vapor deficit [51], may have encouraged higher evaporation from the litter.

## 4. Discussion

### 4.1. Understanding Sprinkler Cooling

Understanding a sprinkler-type surface-wetting system requires a complete rethinking of how to cool chickens and overcoming the initial fear of a slightly elevated house temperature. Sprinkler cooling attempts to cool individual chickens, unlike cool cell systems that attempt to cool the environment where the chickens live. Sprinkler cooling requires a slightly warmer house temperature and an associated lower house humidity other than a standard cool cell system usually provides. This warmer, drier environment does two things: (1) it creates a situation where the bird can more effectively and efficiently cool itself through evaporative respiration, and (2) it creates an auxiliary heat release mechanism, replacing the loss of sensible heat dissipation due to hot weather [31,50,52].

It may surprise some that the sprinkler system did not have a more obvious effect on litter moisture, as one might think applying water directly to the birds would increase litter moisture content. However, the quantity of water reaching the litter from the sprinklers is generally much less than the amount of water that the birds add to the litter in the manure [53]. The quantity of water added to the floor (including onto the birds) by the sprinklers (median 0.07 L/m^2^/day, maximum 1.04 L/m^2^/day) is less than the amount of water the birds add to the litter in their excreta (estimated to be 1.6 L/m^2^/day to 3.3 L/m^2^/day) [51].

In addition, even though the sprinkler system applied greater amounts of water on hotter days, the house was operating in tunnel ventilation mode on these hotter days with maximum air speed from all tunnel fans in operation. These conditions were favorable for rapidly evaporating the water applied to the birds by the sprinklers before it had the opportunity to reach the litter (while also ensuring a maximum evaporative cooling effect). A key feature is to maintain an interval between sprinkler operations that is sufficient to allow the water applied during one sprinkler application to be evaporated just before the next sprinkler application. It is important to allow the birds to dry off between sprinkler applications.

The phase change from liquid to water vapor taking place at the birds’ surfaces is much more efficient at dissipating heat than convective heat transfer between chicken bodies and the warm surroundings [48]. Furthermore, as birds age and more completely fill the house, only a small portion of the water applied by the sprinklers actually reaches the litter due to water droplets landing only on the birds that now cover all the floor area. We did observe a tendency for litter moisture to be slightly drier in the sprinkler/cool cell combination house compared to the cool cell only house. In general, litter moisture content is influenced by multiple factors [39], such as those associated with drinkers, ventilation rate, bird health, and litter properties which were beyond control within the scope of this study.

### 4.2. Sprinkler Advantages

The reason the combination cooling achieved a large amount of cooling water saving lies in the way water is employed. The quantity of water used by a cool cell system is largely determined by the psychrometric process and is a function of the outside temperature and humidity conditions, the amount of heat released by birds, and the amount of ventilation air exchange through the house. High cooling water usage from a cool cell system is a consequence of the high ventilation rate at approximately one air exchange per minute to exhaust humidified air. The quantity of water required to cool birds through surface wetting followed by evaporation is governed by the heat production demand of the birds and the temperature surrounding the birds. Birds’ surface acted as a local evaporative device, hence high efficiency with relatively small and controlled amount of water in achieving birds’ cooling. Even though houses experience the same amount of air exchange under tunnel ventilation, the exhaust air in the combination house was minimally altered and remained similar to the psychrometric condition as the outside air.

Sustainability has become a key focus for the poultry industry in recent years. In addition, water scarcity is a serious concern that can no longer be ignored. Sprinkler cooling of broilers offers considerable water savings without sacrificing flock performance. As the poultry industry seeks opportunities to lessen its environmental footprint and become more sustainable, sprinkler cooling should be considered an important tool to address water scarcity, improve water conservation efforts, and help the industry reach its sustainability goals.

## 5. Conclusions

The combination cooling of heavy broilers used the sprinklers as the first line of cooling defense, with cool cells used only to prevent extreme warm conditions, to best achieve the full potential of sprinkler cooling. The management employed in this study allowed 64% of cooling water saving compared to cool cells alone. The houses with combination cooling were warmer during the daytime period when supplemental cooling by water is necessary, with an average daily maximum temperature of 2.2 °C above that in a house typically operated under a cool cell system. The relative humidity remained lower in the combination houses than in typical cool cell houses. This study indicated that an intermittently operated surface wetting method in tunnel ventilated houses can be effective to compensate for indoor house temperature up to 31.6 °C (89 °F) in cooling floor-raised broiler chickens up to 4.42 kg. Operating the house slightly warmer and drier by delaying the cool cell system is critical in maintaining similar litter quality under the two cooling regimes.

## Figures and Tables

**Table 1:** Summary of days during grow-outs when the cooling systems were used.

Flock #	Harvest Date	Cooling Allowed in Combination House		Cooling Allowed in Cool Cell Only House
		Sprinkler	Cool Cell	
1	20 July 2020	37–61 d	53–61 d	37–61 d
2	5 October 2020	27–61 d	27–61 d	27–61 d

**Table 2:** Temperature and run time settings suggested by sprinkler manufacturer.

Level	Degrees Offset above TT ^+^	Run Time (s)	Idle Time (min)
1	1.1 °C (2 °F)	10	30
2	2.8 °C (5 °F)	20	15
3	4.4 °C (8 °F)	20	5–7

^+^ TT = tunnel temperature set point.

**Table 3:** Set point temperatures (T) of the sprinkler system (SS)/cool cell (CC) used in this study.

Flock Day	Set-Point T (ST)	Tunnel T (TT)	SS Level 1	SS Level 2	SS Level 3	CC-ON T ^†^
27–61	Temperature, °C					
	23.7–15.6	ST + 3.3	TT + 2.2	TT + 3.9	TT + 5.6	TT + 12.2 (ST + 15.6)
Example						
56	16.7	20	25.6	27.2	28.9	32.2

^†^ Cool cell is allowed to be wetted for a short duration of 20 s at a time when CC setpoint temperature is reached in order to prevent substantial decrease of indoor temperature.

**Table 4:** Cumulative cooling water used in the cool cell (CC) only house and the combination house when raising heavy broilers during May–October 2020.

Flock #	Water Used in CC-Only House (L)	Water Used in Combination House (L)	Portion of Water Used by Sprinklers (L)	Water Saved in Combination House
1 ^†^	147,465	50,266	20,138	66%
2 ^‡^	44,342	17,459	8877	61%

^†^ 30 d > 30 °C, including 6 d > 35 °C (daily maximum ambient temperatures). ^‡^ 13 d > 30 °C (daily maximum ambient temperatures).

## Data Availability

Restrictions apply to the availability of these data. Data were obtained in collaboration with a major poultry integrator and are available from the authors only upon permission from the integrator.
